# Remarks upon the Catarrhus Vesicæ of Old Persons

**Published:** 1829-05

**Authors:** 


### CATARRHUS VESICÆ.

*Remarks upon the Catarrhus Vesicæ of old Persons.* Read at the Royal Academy of Sciences. By Dr. Civiale.

Diseases of the genito-urinary organs have for a long time attracted the particular attention of medical practitioners. Still this part of surgery yet requires considerable improvement. These diseases destroy a great number of persons who are advanced in life, and there are, in fact, but few men who have arrived at a certain age, and who have led a sedentary life, who do not feel some affection of the kind. The particular subjects to which I have directed my attention have induced me to study very carefully the genito-urinary organs, and the maladies to which they are obnoxious.

Old men, especially those who have devoted themselves to literary labours, are frequently attacked with chronic inflammation of the bladder, the progress of which is slow and often insidious. It but too frequently happens that such patients do not apply for medical assistance until the time has passed by when any benefit can be expected from the resources of our art.

The majority of surgeons have considered chronic catarrh of the bladder in old subjects to be almost always a fatal disease. This prognosis should not, however, extend to those accidental inflammations of the bladder which may arise from irregularities of diet, the sudden application of cold, or the recession of certain cutaneous affections. In these cases the disease is usually much less severe, and more under the control of remedies. This is also the case when the disease arises from the presence of a foreign body in the bladder, or from some obstacle to the passage of the urine through the urethra. If in such cases the cause be removed, the symptoms disappear.

It is my intention, upon the present occasion, particularly to consider the *catarrhus vesicæ* which arises at an advanced period of life, without any manifest cause, and for which we have yet to devise any satisfactory and effectual mode of treatment. Numerous facts have convinced me that this common and serious disease of the bladder is the result of muscular atony of the viscus. In old age the bladder naturally becomes torpid, and the urine is expelled slowly and with difficulty. This weakness of the bladder is increased by a sedentary life, a sitting posture, the use of soft and warm cushions, and particularly by neglecting the first inclination to make water, and by violent and frequently repeated mental emotion. When the bladder is distended, its contractions are imperfect, and only a part of the urine is evacuated. Hence arises inflammation of the mucous membrane, a collection of mucus takes place, and great irritation is the consequence. The inflammation becomes more severe, extends, and at length invades the whole internal surface of the bladder. The muscular coat is deprived more and more of its power of contracting, like all other muscles which are the seat of inflammation. Notwithstanding the inclination which the patient now feels, it is with much difficulty and pain, and great effort, that he can pass even a small quantity of glairous urine, which is frequently almost solid and of a deep colour. This state may continue for a long time. Various changes may arise from the effects of diet or medical treatment. The disease may even be much alleviated, and a speedy cure be anticipated: but our hopes will be disappointed; the symptoms will reappear with additional severity. The general health becomes deteriorated, the various functions of the body are disturbed, fever arises, marasmus follows, and the patient dies. After these remarks, the nature of the means we should employ must be evident. We must endeavour to diminish the sensibility of the urethra, if the patient is very irritable, to facilitate the flow of urine, to prevent the accumulation and retention of glairy matter in the bladder, to change the morbid action of the viscus, and to transfer the irritation to the exterior of the body. These indications are precise, and not difficult to fulfil. We know that we can always accustom the urethra to the presence of a foreign body, so that the passage of a catheter may be effected without either difficulty or pain. By the assistance of this instrument, the flow of urine and mucous matter is facilitated, and we can also replace the irritating contents of the bladder by mild injections, which may be changed for those of a more tonic nature, in proportion as the irritability of the bladder is diminished. When by these means we have restored the contractile power of the bladder, and have rendered the urine limpid and its evacuation easy, we may have recourse to cold applications applied to the perineum and hypogastrium, and to dry aromatic frictions upon the same parts, and also upon the inferior extremities.

It is almost superfluous to add, that the patient must pay attention to his diet, take moderate exercise, and keep his bowels in regular order. For common beverage he may take soothing aromatic liquids.

It must be presumed that the practitioner will possess sufficient judgment to modify the details of his practice according to the duration and intensity of the disease, the constitution of his patient, and the influence of climate.

Among the numerous facts which convince me of the utility of the curative plan I propose, I may cite the following:

A man, upwards of seventy years of age, had felt for about a year the first symptoms of catarrhus vesicæ, for which he had been treated, in succession, by the best practitioners of France, Switzerland, and Germany. Antiphlogistics, narcotics, derivatives, and various other remedies, had been either alternately or simultaneously employed. The urine, however, became more and more loaded with glairy, fetid, black, and purulent matter; the pain was more severe and continued; the patient lost his appetite and strength, and passed sleepless nights; he was no longer able to leave his room. To the symptoms of catarrhal affection was joined obstinate constipation, the effect of opium, which had been administered in large doses.

I was consulted, and I recommended the patient's removal to Paris. In endeavouring to assure myself that he had no stone in the bladder,. I found that the bladder could not empty itself, that its internal surface was very irritable, and that when the catheter came in contact with it great pain was produced. These circumstances, and the condition of the urine, indicated the existence of an intense and long-standing catarrh. The symptoms had probably been increased by a voyage of 150 leagues.

The remedies previously employed were forthwith abandoned; the patient was placed upon a cold regimen, and ordered to take soothing drinks, and to have emollient clysters. The catheter was introduced merely to facilitate the flow of urine and of the glairy matter, and to inject at first tepid, and then almost cold water. This mode of treatment, in about three weeks, reestablished the contractile power of the bladder, alleviated the pain, and rendered the urine limpid. The patient regained his appetite, and slept better, and was now, in fact, in a very gratifying state.

By the employment of lithotrity, I have been led to the detection of the cause which principally contributes to keep up the catarrhus vesicæ in old persons, and to the treatment the best adapted to oppose the progress of the malady. I have observed that patients affected at the same time with stone and catarrhus vesicæ so severely as to render the operation dangerous, were always much relieved after the first and second attempts to break down the calculus in the bladder. The urine, which had been turbid, glairy, fetid, and purulent, suddenly became as limpid as in the healthy state. The evacuation of it, which had been previously difficult and painful, became easy. The patients, who were debilitated, constantly suffering, and desponding in mind, were more cheerful and animated. In a few days a remarkable improvement was observable in the general health. Such an alteration could not be attributed to the diminished size of the calculus; for experience teaches us that, in strictly calculous diseases, the patient is not relieved until the last fragments of stone are removed. My attention was therefore directed to the particular state of the bladder, and to the influence which the operation exercised upon that organ. I ascertained that most of the patients suffered less from the stone than from the catarrh of the bladder. The painful straining they experienced was when they began to pass their urine, and not when the stream had almost ceased to flow. The introduction of a catheter immediately after the patient had made water proved that the bladder was not entirely emptied; that there was, in fact, a state of torpidity, and some degree of paralysis, of the organ. The presence of the catheter, the frequent evacuation of the urine and mucous matter, and the injection of a sufficient quantity of water to distend the bladder, together with the friction of the branches of the instrument,* changed its morbid action, and restored its contractile power. Hence the improvement which I have pointed out.

The same experiments have been followed by the same results when no stone was contained in the bladder.

These considerations upon a morbid state, connected in so many respects with the disease of stone in the bladder, led me to offer a few remarks upon lithotrity. The results which I last year obtained by the application of this mode of treatment, have been still more satisfactory than those of the preceding years. Patients, whose condition at first prohibited the employment of it, have been operated upon with success, notwithstanding considerable enlargements of the prostate gland, or of fungus, or old standing catarrh of the bladder. So much was the general health disturbed in some of these patients, that several surgeons had refused to perform the operation of lithotomy. I may mention two facts. M. D. came to Paris in 1827. He was admitted into an hospital, and some ineffectual efforts were made to crush the stone. The symptoms produced by these attempts were so severe that they could not be repeated; neither could any other operation be had recourse to. The patient returned home, with the hope of reestablishing his health, but the symptoms of inflammation of the bladder continued. Wearied with suffering, and conscious of gradual exhaustion, M. D. again visited Paris, but it was not yet deemed prudent by other surgeons to attempt either the operation of lithotrity or of lithotomy. The stone was of considerable size, the bladder seriously affected, the general health very bad, and the patient much emaciated. Such were the appearances when I was consulted. The operation† completely succeeded: it was performed at La Pitié, in the presence of M. Lisfranc and his numerous pupils. The patient was dismissed perfectly cured.

The other case is still more remarkable. M. P., of

* M. Civiale refers to the instrument employed in breaking down the stone in the bladder.—Ed.

† Of breaking down the stone in the bladder, in the manner recommended by M. C. and other surgeons.—Ed.

Paris, had been under treatment for a year, sometimes for stricture of the urethra, and sometimes for disease of the prostate, and lastly for ulcers in the bladder. No benefit was obtained from any of the means that had been adopted. I was at length consulted. I ascertained that the bladder contained a large stone, of unequal and rough surface, which had deceived those who had previously examined the patient respecting the nature of his disease. The stone being composed of calcareous phosphat, which was very friable and soft, particularly on its superficies, it was mistaken for a soft body. In other respects M. P. was in the most unfavorable state. The urine was purulent, and flowed away involuntarily; loss of appetite, sleepless nights, continued fever, and great emaciation. The operation was performed in the presence of many practitioners, among whom was M. Dubois. In four sittings, of about five minutes each, the stone was broken down and extracted. No particular symptoms followed, and the patient recovered much more rapidly than could have been anticipated.

As might be expected, the operation of lithotrity has already considerable influence upon the minds of patients afflicted with stone in the bladder. They now begin to be aware that they should lose no time before they submit to its employment. Each year I see about the same number of patients. In 1825 and 1826, I only operated upon about one third of those who presented themselves; in 1827, upon more than one half; in 1828, two thirds were cured by lithotrity. The shorter the duration of the disease, the smaller and less numerous are the calculi: in such cases, therefore, the treatment is much easier and less tedious. In a great number of recent cases, one or two very short sittings have been sufficient to destroy the stone. Thus more than one hundred patients afflicted with stone, and upon whom I have successfully operated in a short space of time, confirm the importance of lithotrity, and the judgment which the Academy has passed upon this mode of treatment.*

